# The moderating effect of age on loneliness and subjective remaining life expectancy among Chinese adults

**DOI:** 10.3389/fpubh.2026.1873655

**Published:** 2026-07-29

**Authors:** Sok Leng Che, Sok Man Leong, Kuai In Tam

**Affiliations:** 1 Nursing and Health Education Research Centre, Kiang Wu Nursing College of Macau, Macau, Macao SAR, China; 2 Research Management and Development Department, Kiang Wu Nursing College of Macau, Macau, Macao SAR, China; 3 Education Department, Kiang Wu Nursing College of Macau, Macau, Macao SAR, China

**Keywords:** age difference, China, life-course differences, loneliness, subjective remaining life expectancy

## Abstract

**Background:**

Subjective Remaining Life Expectancy (SRLE) plays a critical role in shaping late-life decision-making, such as health behaviors, retirement planning, and end-of-life preparation. Although loneliness has been widely documented to exert adverse physical, mental, cognitive, and behavioral consequences, relatively little is known about how loneliness influences SRLE. This study aimed to examine the association between loneliness and SRLE and to determine whether this relationship differs across age groups.

**Methods:**

A cross-sectional online survey was conducted among 969 individuals recruited from Macao, Zhongshan, Guangzhou, and Zhuhai. SRLE was calculated using the life-line question from the Longitudinal Aging Study Amsterdam. Loneliness was measured using the Chinese version of the three-item UCLA Loneliness Scale. Moderation analyses were performed to examine age-group differences in the relationship between loneliness and SRLE.

**Results:**

The mean SRLE was 41.39 years, and the mean loneliness score was 4.54. Loneliness and age were both significantly negatively associated with SRLE. Significant age-group differences were observed, with younger adults (18–44 years) reporting higher loneliness levels and longer SRLE than adults aged 45 years and older.

**Conclusion:**

The association between loneliness and SRLE was observed primarily among younger adults and remains unclear among older adults. This pattern highlights life-course differences in how loneliness relates to perceived remaining lifespan, suggesting that interventions aimed at strengthening social connections may be particularly beneficial for younger adults.

## 1 Introduction

Subjective remaining life expectancy (SRLE) refers to an individual’s perceived number of remaining years of life, and is commonly defined as the difference between one’s current age and the age at which one expects to die (1). Unlike average life expectancy at population-level, which primarily informs macro-level policy and planning, SRLE functions at the micro level by shaping individuals’ decision-making and behavioral choices (2). Individuals adjust their health-related behaviors, medical planning, retirement decisions, and financial arrangements in accordance with their perceived remaining life expectancy (1, 3, 4). Within the fields of health and healthcare, SRLE may serve as a psychological barrier to the adoption of health-promoting behaviors (5), and can be seen as a predictor of mortality risk (6). Empirical evidence indicates that SRLE declines with increasing age, poorer self-rated health, and the presence of chronic or major illnesses (7, 8), suggesting that individuals integrate both age and health status when forming expectations about their remaining lifespan. SRLE is a dynamic construct that evolves over time and is shaped by individuals’ aging trajectories, major life events, health status, and overall well-being (9, 10). Age is a key determinant of subjective life expectancy. Many of the existing research on SRLE has focused on middle-aged and older adults (2, 11). Because during these later stages of the life course, remaining lifetime becomes more salient than time already lived (11). This population is often in the late stages of their careers or transitioning into retirement, and SRLE is closely linked to decisions related to retirement, purchase of age-related services, pension planning, and anticipated long-term care needs (2).

Although SRLE has often been examined in middle-aged and older populations because of its relevance to retirement, healthcare planning, and end-of-life preparation, perceived remaining lifetime is not exclusive to later life. Across adulthood, individuals’ remaining lifespan may shape health behaviors, life planning, decision making, and perceived control over the future (12). Thus, SRLE can be understood as a life-course construct that reflects how people at different ages evaluate their remaining lifespan in relation to their current health, social circumstances, and developmental context. The psychological basis of SRLE may also differ across life stages. Among younger adults, SRLE may reflect perceived future opportunities, health expectations, and general optimism or pessimism about life trajectory, whereas among older adults it may be more closely anchored in current health status, functional capacity, illness experience, retirement planning, and ageing-related concerns (13). Therefore, the use of SRLE across a broad adult age range should be interpreted from a life-course perspective rather than as assuming that younger and older adults construct SRLE in exactly the same way, examining SRLE across a broad adult age range can provide insight into how psychosocial experiences, including loneliness, relate to perceived future time at different stages of adulthood.

Loneliness is a subjective and negative emotional condition that occurs when individuals perceive a discrepancy between the quality or quantity of social relationships they desire and those they actually experience (14). While loneliness may be temporary for some individuals, persistent loneliness has been shown to have a significant risk factor for adverse health and well-being outcomes (15, 16). Both cross-sectional and longitudinal studies have consistently demonstrated that loneliness is associated with a wide range of adverse health outcomes. Therefore, it has emerged as a significant public health challenge (14, 17). Individuals experiencing loneliness tend to report poorer self-rated health (18, 19), lower levels of well-being (20), more unhealthy lifestyle behaviors (21), and higher risk of mortality (22). In terms of physical functioning, loneliness is associated with a higher likelihood of declines in activities of daily living and reduced mobility (23, 24). Regarding mental health, lonely individuals are more likely to experience symptoms of anxiety and depression and show an increased risk of suicidal ideation (19–21). Loneliness has also been linked to subsequent declines in cognitive function over time (25–27). Moreover, persistent loneliness is associated with greater healthcare utilization and an elevated risk of mortality (21, 23, 28). One of the possible mechanisms is that loneliness is a psychological process that alters physiological functioning by heightening individuals’ vigilance to perceived social threats, thereby increasing susceptibility to disease and elevating the risk of mortality (29–31).

Importantly, loneliness should be distinguished from related but conceptually distinct constructs. Social isolation generally refers to the objective absence or limited availability of social network resources or social interaction, whereas loneliness reflects the subjective and unwanted perception that one’s social relationships are insufficient or unsatisfactory (32). Similarly, being alone does not necessarily indicate loneliness, as solitude may be voluntarily chosen and experienced as neutral, restorative, or satisfying by some individuals (33, 34). In the present study, loneliness is therefore conceptualized as unwanted perceived social disconnection rather than as objective isolation or voluntary solitude. Loneliness is associated with increased risks of stroke, heart disease, and all-cause mortality, making it an urgent and significant public health concern (22, 35, 36). Epidemiological evidence on age differences in loneliness is heterogeneous. Some global and population-based studies suggest that loneliness may be more commonly reported among younger and middle-aged adults than among older adults, whereas other studies have found higher loneliness in later life or non-linear patterns across the life course (14, 21). For example, the World Health Organization estimated loneliness prevalence at 15.1% among adults aged 30–59 years and 11.8% among adults aged 60 years and older (14). However, age patterns in loneliness vary across cultural, social, health, and methodological contexts (37). Thus, loneliness should not be framed exclusively as a problem of later life; rather, it may affect adults at different life stages in different ways.

Although loneliness has been widely documented to exert adverse physical, mental, cognitive, and behavioral consequences, its relevance to SRLE has received limited attention. Theoretically, loneliness may shape SRLE because it influences how individuals evaluate their health, social resources, future orientation, and vulnerability to mortality-related risk. Loneliness is associated with poorer self-rated health, lower well-being, depressive symptoms, heightened vigilance to perceived social threat, and increased mortality risk, all of which may contribute to more pessimistic expectations about one’s remaining lifetime. In this sense, SRLE can be understood not only as an estimate of expected longevity but also as a subjective appraisal that integrates perceived health, psychosocial resources, and expectations about the future. Because the meaning and determinants of SRLE may vary across adulthood, examining whether age moderates the association between loneliness and SRLE can help clarify whether loneliness is similarly relevant to perceived remaining lifetime at different life stages. Accordingly, the present study aims to examine the association between loneliness and SRLE and to determine whether this relationship differs across age groups.

## 2 Methods

### 2.1 Design and sampling

This study adopted a cross-sectional survey design. Participants were recruited using convenience and snowball sampling from four cities: Macao, Zhongshan, Guangzhou, and Zhuhai. The inclusion criteria were: (1) aged 18 years or above; (2) residence in Macao, Zhongshan, Guangzhou, and Zhuhai; (3) self-identification as Chinese; and (4) ability to understand the informed consent and questionnaire content. Participants were excluded if they reported cognitive impairment or severe mental health conditions that could substantially affect their ability to comprehend the survey questions, provide valid self-reported responses, or give informed consent. Participants who did not provide informed consent or who withdrew consent during the study were also excluded.

### 2.2 Data collection and instruments

An online structured questionnaire was disseminated through advertisements, social media platforms, and local social service organizations. Data were collected using the online survey platform Wen Juan Xing^1^ from August to December 2025. The first page of the questionnaire provided a comprehensive description of the study. Subsequent pages required participants to indicate their agreement with statements serving as the informed consent form. Participants were unable to proceed with the questionnaire without providing consent. Those who selected “disagree” were redirected to a thank-you page and excluded from participation. After providing informed consent, participants were asked two screening questions to verify their eligibility: whether they resided in one of the four study cities (Macao, Zhongshan, Guangzhou, or Zhuhai) and whether they were aged 18 years or above. Only respondents who answered “yes” to both questions were allowed to proceed to the questionnaire. The questionnaire was initiated only after informed consent had been obtained. To reduce the possibility of duplicate responses, the survey was configured so that each IP address could submit the questionnaire only once. After data collection was completed, all responses were manually screened for data quality. Responses showing obvious response patterns or clear inconsistencies were treated as invalid and excluded from the analysis.

The questionnaire comprised three sections: (1) subjective remaining life expectancy (SRLE), (2) loneliness, and (3) participants’ sociodemographic characteristics and health status. To assess its applicability, a pilot study was conducted with 30 participants from the target population. No modifications were required following analysis of the pilot results.

*Subjective remaining life expectancy* (SRLE) was measured using the Longitudinal Aging Study Amsterdam (LASA) life-line question developed in the Longitudinal Aging Study Amsterdam. This question assesses respondents’ subjective expectations regarding their remaining lifespan by capturing the number of years they believe they are likely to live (38). SRLE was assessed using a graphical lifetime measure. In the original version, a “lifeline” was presented to participants on paper (1, 38). The lifeline consisted of a 25-cm horizontal line, with the word “beginning” placed at the far left and the word “end” placed at the far right. Participants were instructed that the line represented their lifespan and were asked to mark the point on the line where they felt they were currently standing in their life with a cross. Due to the limitations of the online questionnaire format, it was not possible to use a cross mark to indicate participants’ perceived current position in their lifespan. Therefore, we adapted the measure by using the slider function in the online questionnaire. Participants were asked “The line below represents the beginning (0) and the end (25) of your life journey. Please slide to where you feel you are in your life right now.” The slider similarly displayed 25 intervals to represent the participants’ lifespan. SRLE was calculated by subtracting the respondent’s current age from their subjective life expectancy (SLE). SLE was derived by dividing the respondent’s current age by the proportion of the life-line that they perceived as already lived. Respondents with an estimated SLE of 150 years or older were excluded from the analysis, as such values were considered indicative of a potential misunderstanding of the life-line question (1). Higher SRLE scores indicate that participants perceived themselves as having a longer remaining lifespan.

*Loneliness* was assessed using the Chinese version of the three-item UCLA Loneliness Scale (39). The UCLA three-item Loneliness Scale has been validated in large-scale population surveys across multiple countries, demonstrating good validity in detecting loneliness. The scale assesses loneliness by asking about the frequency of related feelings without explicitly using the terms “loneliness” or “feeling lonely.” It consists of three items: (1) “How often have you felt that you lacked companionship in the past two weeks?” (2) “How often have you felt left out in the past two weeks?” and (3) “How often have you felt isolated from others in the past two weeks?” Responses are rated on a four-point Likert scale ranging from 0 (“never”) to 3 (“often”), yielding a total score between 0 and 9, with higher scores indicating greater loneliness. The original scale reported a Cronbach’s *α* of 0.72, while in the present study it was 0.88. In addition to treating loneliness as a continuous variable, loneliness was categorized using the established cut-off (40). Those with a total score of 0 to 5 were categorized as “not lonely,” and those with a score of 6 to 9 were categorized as “lonely.”

The section of demographic information collected participants’ sociodemographic information, including gender, age, educational level, marital status, number of children, religion, employment status, living status, self-rated health, and the presence of chronic diseases. Age was categorized into two groups, 18–44 years and ≥45 years, for the moderation analysis. This categorization was adopted primarily to maintain sufficient subgroup size because the number of participants aged ≥45 years was relatively small (*n* = 161) compared with those aged 18–44 years (*n* = 808). Further subdivision of the older age group would have resulted in small subgroup sizes and potentially unstable estimates. Therefore, the age-group analysis should be interpreted as a broad comparison between younger adults and middle aged and older adults rather than as a examination of age-related trajectories across adulthood.

### 2.3 Data analysis

No missing data was identified in the dataset because all items in the online questionnaire were mandatory, and participants could submit the questionnaire only after completing all questions. Participants who discontinued the questionnaire before submission were not recorded in the database. Descriptive statistics, including means, standard deviations, frequencies, and percentages, were used to summarize participants’ characteristics. Independent-samples t tests were conducted to examine differences in SRLE across participant characteristics. The association between loneliness and SRLE was assessed using Pearson correlation analysis. Multivariate linear regressions were performed to identify factors associated with SRLE and to test the interaction effect between loneliness and age groups on SRLE. Moderation analyses were conducted using Hayes’ PROCESS macro (version 5.0) in SPSS (41) with 5,000 bootstrap samples, controlling for gender, educational level, marital status, whether they have children, religion, living status, employment status, self-rated health, and presence of chronic diseases. Statistical significance was set at *p* < 0.05. All data management and statistical analyses were performed using IBM SPSS Statistics version 30.0.

### 2.4 Ethical approval

This study was reviewed and approved by the Ethics Committee of Kiang Wu Nursing College of Macau (ID: REC-2024.0704). All procedures in this study were performed in accordance with the Declaration of Helsinki. The participants provided their informed consent to participate in this study.

## 3 Results

### 3.1 Participant characteristics

The survey approached 1,449 potential participants, received 1,125 responses, the response rate was 77.6%. Among 1,125 responses, 156 were excluded for the following reasons: unwillingness to participate at the start of the questionnaire (*n* = 19), residence outside Macao, Zhongshan, Guangzhou, or Zhuhai (*n* = 41), age below 18 years (*n* = 22), obvious response patterns (*n* = 4), and invalid SRLE responses (*n* = 70). A total of 969 valid responses were therefore included in the final analysis. The majority of participants were female (57.28%) and aged 18–44 years (83.38%), with 78.33% holding a bachelor’s degree or higher in education. Approximately half of the participants were not married or cohabiting (50.67%), and more than half did not have children (54.80%). Most participants reported having a religious belief (73.07%), living with others (78.74%), and being engaged in paid employment (76.78%). In terms of health status, 57.89% rated their health as fair or poor, and 77.09% reported no chronic diseases. About one-third of participants were lonely (36.95%). SRLE was observed longer among participants aged 18–44 years, those with an education level of bachelor’s degree or above, those reporting good or very good self-rated health, those without chronic diseases, and those who were loneliness. No significant differences in SRLE were observed across other sociodemographic characteristics (Table 1).

**Table 1:** Correlation between participant characteristics and SRLE.

Participant characteristics	n	%	Mean	SD	*t*	*p-value*
Total	969	100.00	41.39	26.96		
Gender					−1.37	0.172
Male	414	42.72	40.02	26.89		
Female	555	57.28	42.41	26.99		
Age (years)					3.06	0.002
18–44	808	83.38	42.57	27.31		
> = 45	161	16.62	35.48	24.35		
Education level					−2.37	0.018
Below bachelor’s degree	210	21.67	37.50	26.53		
Bachelor’s degree or above	759	78.33	42.47	26.99		
Marital status					1.70	0.090
Not married or cohabiting^	491	50.67	42.84	28.51		
Married or cohabiting	478	49.33	39.91	25.21		
Children					1.63	0.104
None	531	54.80	42.66	28.11		
Yes	438	45.20	39.86	25.44		
Religion					1.23	0.218
No	261	26.93	43.15	26.95		
Yes^†^	708	73.07	40.74	26.95		
Living alone					0.51	0.613
No	763	78.74	41.62	27.16		
Yes	206	21.26	40.55	26.23		
Employment					0.41	0.683
No	225	23.22	42.09	30.10		
Yes	744	76.78	41.18	25.95		
Self-rated health					−5.36	<0.001
Fair/bad/very bad	561	57.89	37.39	24.48		
Good/very good	408	42.11	46.90	29.17		
Chronic disease					3.30	0.001
None	747	77.09	42.86	27.40		
Yes	222	22.91	36.44	24.85		
Loneliness					5.06	<0.001
Not lonely	611	63.05	44.70	27.29		
Lonely	358	36.95	35.74	25.45		

SD, standard deviation.

^includes not married, separated or divorced, and widowed.

†includes Catholicism, Christianity, Taoism, Buddhism, folk beliefs, and others.

### 3.2 Descriptive statistics and correlation analysis

The mean loneliness score was 4.54 ± 2.44, and the mean SRLE was 41.39 ± 26.96. Loneliness was significantly and negatively correlated with SRLE (*r* = −0.211, *p* < 0.001). Among participants aged 18–44 years, the mean loneliness score was 4.66 ± 2.40 and the mean SRLE was 42.57 ± 27.31. A significant negative correlation between loneliness and SRLE was also observed in this age group (*r* = −0.247, *p* < 0.001). In contrast, among participants aged 45 years and older, the mean loneliness score was 3.93 ± 2.54 and the mean SRLE was 35.48 ± 25.35, with no significant association between loneliness and SRLE (Table 2).

**Table 2:** Descriptive statistics and correlation coefficients.

Variables	Total		Aged 18–44		Aged ≥45		*t*	*p*-value
	mean	SD	mean	SD	mean	SD		
Loneliness	4.54	2.44	4.66	2.40	3.93	2.54	3.47	<0.001
SRLE	41.39	26.96	42.57	27.31	35.48	24.35	3.06	<0.001
Pearson correlation (*r*)	−0.211***		−0.247***		−0.107			

***p < 0.001.

### 3.3 Results of moderation analysis

Table 3 presents the results of the regression analyses examining whether the association between loneliness and SRLE differed between the two predefined age groups. In Model 1, the main effect of loneliness on SRLE was statistically significant [B = −2.07, *p* < 0.001, 95% CI (−2.80, −1.34)], indicating that higher levels of loneliness were associated with lower SRLE across the full sample. A significant main effect of age was also observed [B = −7.85, *p* = 0.003, 95% CI (−13.04, −2.67)], suggesting that adults aged 45 years and older reported substantially lower SRLE compared with those aged 18–44. Additionally, the interaction term of loneliness and age in Model 2 was statistically significant [B = 1.86, *p* = 0.039, 95% CI (0.09, 3.62)], indicating that the strength of the association between loneliness and SRLE differed between the two age groups. In supplementary analyses (Table 4) using the established loneliness categorization (not lonely vs. lonely), the interaction between loneliness and age group was no longer statistically significant [B = 9.18, *p* = 0.05, 95% CI (−0.17, 18.54)] (Model 4 in Table 4). However, the direction of the association remained consistent with that observed in the primary analysis treating loneliness as a continuous variable.

**Table 3:** Multiple linear regression of SRLE with loneliness as continuous variable.

Variables	M1							M2						
	95% CI							95% CI						
	B	SE B	*β*	*t*	Lower	Upper	VIF	B	SE B	*β*	*t*	Lower	Upper	VIF
Constant	52.95	3.92		13.50	45.25	60.65		54.43	3.98		13.67	46.62	62.25	
Gender (ref.: Male)														
Female	1.96	1.74	0.04	1.13	−1.44	5.37	1.06	2.25	1.74	0.04	1.29	−1.16	5.66	1.06
Age (years) (ref.:18–44)														
≥45	−7.85	2.64	−0.11	−2.97**	−13.04	−2.67	1.39	−15.46	4.53	−0.21	−3.41***	−24.35	−6.56	4.10
Education level (ref.: below bachelor’s degree)														
Bachelor’s degree or above	1.85	2.15	0.03	0.86	−2.38	6.07	1.13	1.86	2.15	0.03	0.87	−2.35	6.08	1.13
Marital status (ref.: not married or cohabiting)														
Married or cohabiting	−4.52	2.53	−0.08	−1.79	−9.48	0.43	2.29	−4.40	2.52	−0.08	−1.74	−9.35	0.55	2.29
Children (ref.: none)														
Yes	1.01	2.58	0.02	0.39	−4.06	6.08	2.37	0.99	2.58	0.02	0.38	−4.07	6.05	2.37
Religion (ref.: no)														
Yes	−2.32	1.96	−0.04	−1.19	−6.16	1.52	1.08	−2.27	1.95	−0.04	−1.16	−6.10	1.57	1.08
Living alone (ref.: no)														
Yes	−1.80	2.27	−0.03	−0.79	−6.25	2.65	1.23	−1.46	2.27	−0.02	−0.64	−5.91	3.00	1.24
Paid job (ref.: no)														
Yes	−1.80	2.09	−0.03	−0.86	−5.91	2.30	1.12	−2.06	2.09	−0.03	−0.98	−6.17	2.05	1.12
Self-rated health (ref.: fair/bad/very bad)														
Good/very good	5.53	1.86	0.10	2.98**	1.88	9.18	1.21	5.49	1.86	0.10	2.96**	1.84	9.13	1.21
Chronic disease (ref.: no)														
Yes	−2.29	2.11	−0.04	−1.08	−6.43	1.85	1.13	−2.29	2.11	−0.04	−1.09	−6.43	1.84	1.13
Loneliness	−2.07	0.37	−0.19	−5.54***	−2.80	−1.34	1.19	−2.41	0.41	−0.22	−5.91***	−3.21	−1.61	1.42
Loneliness *Age								1.86	0.90	0.12	2.06*	0.09	3.62	3.74
*R* ^2^							0.08							0.09
Adj. *R*^2^							0.07							0.07
*F*							7.67***							7.41***
df							(11, 957)							(12, 956)

**Table 4:** Multiple linear regression of SRLE with loneliness as categorial variable.

Variables	M3							M4						
	95% CI							95% CI						
	B	SE B	*β*	*t*	Lower	Upper	VIF	B	SE B	*β*	*t*	Lower	Upper	VIF
Constant	46.07	3.59		12.83	39.03	53.12		46.41	3.59		12.93	39.37	53.46	
Gender (ref.: male)														
Female	2.12	1.75	0.04	1.21	−1.32	5.55	1.06	2.31	1.75	0.04	1.32	−1.12	5.75	1.06
Age (years) (ref.:18–44)														
≥45	−7.13	2.66	−0.10	−2.68	−12.35	−1.90	1.38	−10.42	3.16	−0.14	−3.30	−16.62	−4.22	1.96
Education level (ref.: below bachelor’s degree)														
Bachelor’s degree or above	1.75	2.17	0.03	0.81	−2.51	6.01	1.13	1.85	2.17	0.03	0.85	−2.41	6.10	1.13
Marital status (ref.: not married or cohabiting)														
Married or cohabiting	−4.00	2.55	−0.07	−1.57	−9.00	0.99	2.28	−3.76	2.54	−0.07	−1.48	−8.75	1.23	2.29
Children (ref.: none)														
Yes	1.22	2.61	0.02	0.47	−3.91	6.34	2.38	1.24	2.61	0.02	0.48	−3.88	6.35	2.38
Religion (ref.: No)														
Yes	−3.24	1.96	−0.05	−1.65	−7.10	0.61	1.07	−3.19	1.96	−0.05	−1.63	−7.04	0.66	1.07
Living alone (ref.: no)														
Yes	−1.74	2.29	−0.03	−0.76	−6.23	2.76	1.24	−1.33	2.29	−0.02	−0.58	−5.83	3.18	1.25
Paid job (ref.: No)														
Yes	−2.25	2.11	−0.04	−1.07	−6.38	1.89	1.12	−2.47	2.11	−0.04	−1.17	−6.61	1.67	1.12
Self-rated health (ref.: fair/bad/very bad)														
Good/very good	6.99	1.84	0.13	3.80	3.38	10.61	1.17	6.92	1.84	0.13	3.76	3.31	10.53	1.17
Chronic disease (ref.: no)														
Yes	−2.44	2.13	−0.04	−1.15	−6.62	1.73	1.13	−2.48	2.12	−0.04	−1.17	−6.65	1.69	1.13
Loneliness (ref.: not lonely)														
Lonely	−6.97	1.84	−0.12	−3.78	−10.59	−3.35	1.12	−8.48	2.00	−0.15	−4.24	−12.40	−4.55	1.32
Loneliness *age							9.18	4.77	0.08	1.93	−0.17	18.54	1.75	
*R* ^2^							0.07						0.07	
Adj. *R*^2^							0.05						0.06	
*F*							6.10***						5.91**	
df							(11, 957)						(12, 956)	

**Figure 1:**
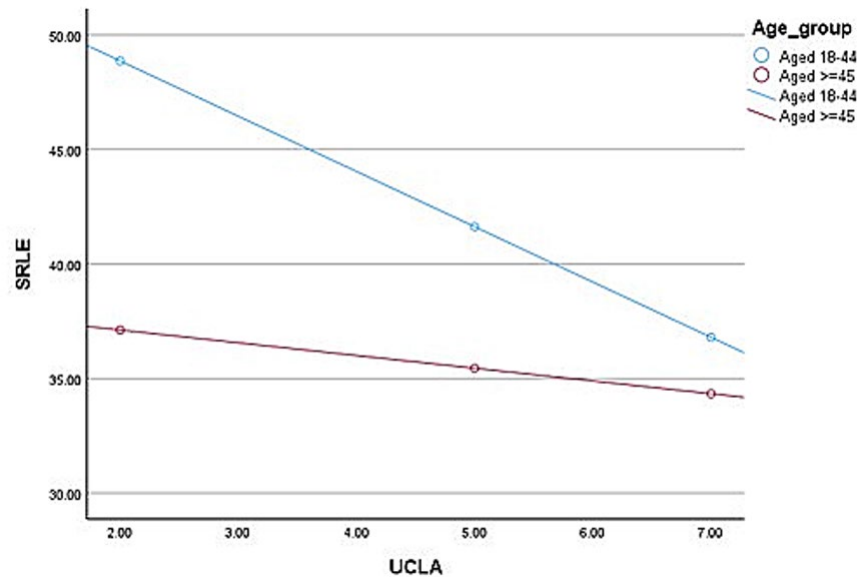
Simple slope analysis of age on loneliness and SRLE.

This interaction was statistically significant both before (Δ*R*^2^ = 0.004, *p* = 0.048) and after (Δ*R*^2^ = 0.004, *p* = 0.039) adjusting for demographic characteristics and health status (Table 5), confirming that age moderates the relationship between loneliness and SRLE. However, the increase in explained variance by the interaction was small, indicating that the interaction accounted for only a limited additional proportion of variance in SRLE. The result of conditional effect (Table 6) showed that loneliness was negatively associated with SRLE among participants aged 18–44 years, whereas this association was not statistically significant among participants aged ≥45 years (Figure 1).

**Table 5:** Moderation analysis of loneliness and age on SRLE.

95% CI											
Model	B	SE	*t*	*p*	Lower	Upper	Δ*R*^2^	*F*	df1	df2	*p*-value
Model 1							0.004	3.921	1	956	0.048
Constant	55.64	2.01	27.70	<0.001	51.70	59.59					
Loneliness	−2.81	0.38	−7.32	<0.001	−3.56	−2.05					
Age	−16.12	4.30	−3.75	<0.001	−24.56	−7.68					
Loneliness *Age	1.78	0.90	1.98	0.048	0.02	3.55					
Model 2							0.004	4.253	1	956	0.039
Constant	54.43	3.98	13.67	<0.001	46.62	62.25					
Loneliness	−2.41	0.41	−5.91	<0.001	−3.21	−1.61					
Age	−15.46	4.53	−3.41	<0.001	−24.35	−6.56					
Loneliness *Age	1.86	0.90	2.06	0.039	0.09	3.62					

Model 1, loneliness, age, and interaction term, unadjusted.

Model 2, loneliness, age, and interaction term, adjusted for gender, education level, marital status, children, religion, living status, employment, self-rated health and chronic disease.

**Table 6:** The conditional effects of age on loneliness and SRLE.

95% CI						
Age group	Effect	SE	*t*	*p*-value	Lower	Upper
Aged 18–44	−2.413	0.408	−5.908	<0.001	−3.214	−1.611
Aged ≥45	−0.556	0.824	−0.676	0.500	−2.173	1.060

## 4 Discussion

This study examined the association between loneliness and subjective remaining life expectancy (SRLE) and whether this association differed by age group. The results suggested that loneliness is associate with individuals’ subjective perceptions of remaining lifetime, but that this association may not operate uniformly across adulthood. In particular, the negative association between loneliness and SRLE was observed primarily among adults aged 18–44 years, whereas it was not statistically evident among participants aged 45 years and older in the present sample. The findings in this study extend previous SRLE research by highlighting loneliness as a psychosocial factor that may shape perceived remaining lifetime. The observed age-group differences in loneliness are consistent with growing evidence that loneliness is not limited to later life. Although loneliness has traditionally been framed as a concern among older adults, recent studies have reported a higher prevalence of loneliness among younger adults (14, 42, 43). Other studies have found higher loneliness in older populations (44) or non-linear patterns across the life course (45, 46). These inconsistencies may be attributable to the dynamic nature of loneliness across the life-course. Loneliness is shaped by a range of life-course–related factors, including exposure to major life events, current health status, perceived social support, and living area (10, 47–50), all of which can influence individuals’ subjective experiences of loneliness. Also, participants with better self-rated health reported longer SRLE in our study, which is consistent with findings from previous research (38, 42). This association may be explained by the fact that individuals who perceive their health as poor are more likely to be reminded of the finiteness of life, thereby forming more pessimistic expectations about their remaining lifespan and reporting shorter SRLE (38).

The relatively low loneliness level observed in this study should also be interpreted in relation to participants’ living arrangements and the distinction between loneliness and social isolation. Loneliness is a subjective perception of insufficient or unsatisfactory social relationships, whereas social isolation refers more to the objective absence or limited availability of social contacts, interactions, or social network resources (32). Although these constructs are related, they are not equivalent. Individuals may live with others yet still feel lonely, while others may live alone without experiencing loneliness (34). In the present sample, most participants were living with others (78.7%), suggesting that many respondents may have had at least some household-level social contact or support. This may have contributed to the relatively low mean loneliness score. However, because living status is only one of the indicators of objective social connectedness and does not capture relationship quality, emotional closeness, perceived support, or frequency of meaningful interaction, the findings should not be interpreted as evidence that co-residence necessarily protects against loneliness. Future studies should assess both loneliness and social isolation using multidimensional measures to better clarify their independent and combined associations with SRLE.

Both loneliness and age had significant negative effects on SRLE. This study demonstrated that higher levels of loneliness were associated with shorter SRLE. This finding is consistent with prior research by Ayalon and colleagues (51), which showed that increases in loneliness and depressive symptoms are associated with an older subjective age. These findings indicate that heightened loneliness may contribute to more pessimistic perceptions of one’s remaining lifespan, as reflected in the observed reduction in SRLE. Importantly, this study further revealed that age moderates the relationship between loneliness and SRLE, with the strength of the association varying across age groups. The negative effect of loneliness on SRLE was significantly stronger among younger adults aged 18–44 years, whereas no significant association between loneliness and SRLE was observed among participants aged 45 years and older. The absence of a significant association may be partly explained by the greater availability of social and healthcare resources in later life. Previous research suggests that individuals who hold more concrete and structured expectations about their future tend to report longer SRLE (13, 52). Compared with younger adults, older adults generally experience lower levels of life uncertainty and benefit from more comprehensive social protection systems and age-targeted services (53). In contrast, younger adults often face higher uncertainty and weaker social safety nets (54). Under conditions of uncertainty, individuals tend to attend more closely to negative information (55). Consequently, when younger individuals experience loneliness in the absence of sufficient formal or informal support, its adverse effects on perceptions of remaining lifespan may be more pronounced than those observed among older adults. Increased investment in both formal and informal support systems may therefore help buffer the effects of adverse psychosocial experiences. Resilience may also play an important role in explaining this finding. Previous research has shown that, during the COVID-19 pandemic, older adults were able to maintain relatively high levels of resilience despite experiencing loneliness due to reduced social interactions, thereby attenuating the negative impact of loneliness on mental health outcomes (56). This adaptive capacity may similarly help buffer the effects of loneliness on SRLE in later life. To enhance the clinical and public health interpretability of the findings, we conducted supplementary analyses using the established categorical classification of the UCLA three item Loneliness Scale. Although the direction of the association between loneliness and SRLE remained consistent, the interaction between loneliness and age group was no longer statistically significant. This discrepancy may reflect the loss of information and reduced statistical power associated with dichotomizing continuous measures. Previous methodological literature has noted that categorizing continuous variables can attenuate associations and reduce the ability to detect interaction effects (57). Therefore, the continuous loneliness score likely provides a more sensitive assessment of age-related differences in the association between loneliness and SRLE. Nevertheless, the categorical analysis offers a clinically interpretable perspective and allows comparison with previous studies using the established loneliness classification.

Because SRLE is age-dependent and may be interpreted differently across adulthood, SRLE scores were interpreted as subjective appraisals of remaining lifetime rather than as objective estimates of longevity. The use of SRLE across participants aged 18–65 years was therefore considered exploratory, and age was examined as a moderator to assess whether the association between loneliness and SRLE differed between age groups. However, although SRLE captures individuals’ subjective appraisal of remaining lifetime, the determinants of this appraisal may differ between younger and older adults (13). As a result, SRLE may not be conceptually equivalent across age groups. This heterogeneity should be considered when interpreting the moderation findings, and future studies should examine whether the measurement meaning and predictors of SRLE vary across different stages of adulthood.

Although the interaction between loneliness and age group was statistically significant, it explained only a small additional proportion of variance in SRLE (Δ*R*^2^ = 0.004). In addition, the overall loneliness level in the sample was relatively low. Thus, the age-group difference in the loneliness–SRLE association should be viewed as preliminary and modest rather than as strong evidence of a robust age-dependent pattern. Nevertheless, the study contributes to the literature by extending SRLE research beyond later life and by suggesting that loneliness may be relevant to perceived future time among younger adults. From a public health perspective, efforts to reduce loneliness should not be limited to older populations. Strategies that strengthen social connectedness, promote meaningful engagement, and support the development of life goals may be beneficial for younger adults, particularly those experiencing social disconnection or uncertainty about the future.

This study has several limitations. First, the cross-sectional design limits the ability to draw causal conclusions regarding the relationship between loneliness and SRLE and does not capture potential changes in these variables over time. Longitudinal studies are needed to examine the causal direction of this association and its dynamic effects across adulthood. Second, participants were recruited using non-probability sampling methods (i.e., convenience and snowball sampling) through an online survey, which may have introduced selection bias and limited the representativeness of the sample, particularly among individuals with limited internet access or digital literacy. Third, the exclusion of individuals with cognitive impairment or severe mental health conditions may limit the representativeness and generalizability of the findings, particularly because these populations may experience loneliness and perceptions of remaining life expectancy differently. Consequently, the sample may not fully represent the broader adult population in the study regions, and the generalizability of the findings should therefore be interpreted with caution. Fourth, the sample was drawn from Macao and three cities in Guangdong Province, e.g., Zhongshan, Guangzhou, and Zhuhai. Given existing urban–rural disparities in income levels, infrastructure development, and access to healthcare resources within the region, the findings may not be generalizable to rural populations. Future research should include cities and regions at different levels of urbanization to further examine the impact of urbanization on SRLE. Finally, the participants were relatively young and with higher educational attainment, with a smaller proportion of participants aged 45 years and older. The dichotomization of age into 18–44 years and ≥45 years was adopted to maintain sufficient subgroup size but may have reduced statistical information and obscured more nuanced age-related patterns. SRLE may not have the same psychological meaning across life stages. Future studies should use larger, more age-balanced samples, refined age modelling, and longitudinal designs to examine whether the predictors and meaning of SRLE vary across the adult life course. Despite several limitations, this study offers a novel perspective by showing that the association between loneliness and subjective remaining life expectancy varies across age groups. The public health emphasis should therefore be placed on strategies aimed at reducing loneliness among younger adults by enhancing social connectedness, promoting engagement in social activities, and supporting the development of life goals and a sense of meaning.

## 5 Conclusion

Age, self-rated health, and loneliness were significantly associated with SRLE. The association between loneliness and SRLE was observed primarily among participants aged 18–44 years, whereas this association was not statistically evident among participants aged ≥45 years in the present sample. This pattern highlights loneliness should not be addressed only as an issue of later life. Public health strategies should include younger adults by strengthening social connectedness, expanding accessible community and mental health support, and promoting meaningful social participation, life planning, and future-oriented resilience. Future longitudinal studies with larger and more age-balanced samples are needed to clarify how loneliness influences SRLE across adulthood and to inform age-sensitive interventions.

## Data Availability

The raw data supporting the conclusions of this article will be made available by the authors, without undue reservation.

## Footnotes

1
https://www.wjx.cn
